# A Prevalence Study of Hearing Loss among Primary School Children in the South East of Iran

**DOI:** 10.1155/2013/138935

**Published:** 2013-07-01

**Authors:** Aqeel Absalan, Ibrahim Pirasteh, Gholam Ali Dashti Khavidaki, Azam Asemi rad, Ali Akbar Nasr Esfahani, Mohammad Hussein Nilforoush

**Affiliations:** ^1^Audiology Department, Faculty of Rehabilitation, Zahedan University of Medical Sciences & Health Services, Zahedan, Iran; ^2^Otolaryngology (ENT) Department, Zahedan University of Medical Sciences & Health Services, Zahedan, Iran; ^3^Department of Anatomical Sciences, Shahid Beheshti University of Medical Sciences, Tehran, Iran; ^4^Audiology Department, Faculty of Rehabilitation, Tehran University of Medical Sciences & Health Services, Tehran, Iran; ^5^Audiology Department, Faculty of Rehabilitation, Isfahan University of Medical Sciences, Isfahan, Iran

## Abstract

Hearing impairment substantially affects child's ability to normally acquire the spoken language. Such negative effects create problems for the child not only in terms of communication but also in terms of achievement in school as well as social and emotional growth. The aim of this research is to study the prevalence of hearing disorders and its relationship to age and gender among primary school students of Zahedan, Iran. In this cross-sectional and descriptive analytical study, 1500 students from elementary schools were screened for hearing loss. The selection of samples was performed using multistage sampling method. Primary information was obtained through direct observation, otoscopy, and audiometric and tympanometric screenings. Data was obtained and analyzed via ANOVA test. Statistical analysis showed a significant correlation between the age and the prevalence of middle ear abnormal function. Conductive hearing loss in males and females was 8.8% and 7.1%, respectively. In addition, 1% and 0.7% of male and female students, respectively, suffered from sensorineural hearing loss. Results indicated that 20.2% of students of elementary schools in Zahedan needed medical treatment for their problems. Therefore, it is recommended that the hearing screening of school-age children should be included in annual school health programs in this region.

## 1. Introduction

Hearing loss of even 15 dBHL can create hearing disability in children and consequently impairment in their mental growth [1–3]. Due to the occurrence of secreted middle ear otitis during a critical period (when the senses are emerging and adapting to the environment), these impairments can create various disabilities in children. These disabilities can cause behavioral complications in six functional areas: mental maturity, perception, speech and speaking, cognition and general intelligence, academic achievement, and interpersonal behaviors [4, 5]. One of the other impairments is unilateral hearing loss (UHL) that, if not examined, is normally detected later because one of the ears is healthy. For the impact of unilateral hearing loss on children's academic achievement, it was found that 30% of children with unilateral deafness lag at least 1.2 years behind their normal peers in terms of academic achievement [6]. Unilateral hearing loss has remarkable effects on academic achievement, language development, and children's auditory perception [7]. By considering the unpredictable difficulties, the best way to identify them would be individual assessment of children at risk. Due to the lack of knowledge and low cooperation of many parents, one of the appropriate times for prognosis, so-called screening, is the school age, because, at this age, the majority of children gather in academic centers and they can all be examined. According to the mentioned subjects and reasons, the Speech and Language Association of America (ASHA) has provided the following guidelines for screening [8].

- The program should be run annually for children aged 3–9 years.
- After nine years of age, the program should be performed annually for children at risk.

## 2. Subjects and Methods

In this cross-sectional and descriptive analytical study, 1,500 students from 30 elementary schools of Zahedan in the academic year 2010-2011 were screened for hearing loss. The selection of samples was performed through multistage sampling method. Out of 146 elementary schools in Zahedan, 30 schools were selected as follows: the schools of the city of Zahedan were divided into six regions based on two educational districts and 3 municipalities, and some schools were randomly selected from each region based on the relative frequency of the schools of that region. From each school, 50 students in 5-year range (6 to 10 years old) were selected randomly (10 persons from each age). In total, 300 males and females per age were included in the study. Physical environment in schools such as noise levels (inside and outside classes) was within the permissible level, and according to ANSI S12.602002, and weather temperature during testing was between 28 and 33°C. Primary information was obtained through direct observation, otoscopic examination, and audiometric and tympanometric screenings of every student. The rejected subjects were referred to for the full examination of hearing system that included complete medical history, pure tone and speech audiometric tests, and tympanometric as well as acoustic reflex assessments. The inclusion criteria for the specialized evaluation included the following.

- Presence of any structural and anatomic problems of auricle and the external ear canal.
- Detection of abnormal cases of the ear canal and eardrum at the time of otoscopic examination.
- No response of either ear to at least one of the experimental frequencies.
- Detection of type B or C tympanogram.

In this study, the MT10 Audiometry and Tympanometry screening device was used for primary screening. The research results were collected by special forms, and the frequency distribution of different hearing impairments was obtained. Chi-square test was also used to compare the differences between both genders, and relationship between hearing impairment and students' age was evaluated by ANOVA test.

## 3. Results

The results of direct observation and otoscopic examination of students are presented in Table 1. The presence of excessive cerumen in canal in this stage of screening was the most common disorder in both genders and in all grades, as the prevalence of this disorder was 8.7% in boys and 7.7% in girls. This difference was not statistically significant. In total, 8.2% of students suffered from this disorder. In tympanometric assessment, type C tympanogram was the most common disorder in all age groups and in both genders, whereas it was prevalent in 11.7% of boys and 6% of girls. The obtained results in this study suggested the influence of gender on the incidence of type C tympanogram, whereas the incidence of these disorders was more prevalent in boys (*z* = 3.9057). Other notable result of the tympanometry screening was the significant decrease of negative pressure incidences in the ear with an increase in age (Table 2). Using Spearman's correlation coefficient, a significant correlation was observed between school grade and the percentage of type C tympanogram (*P* = 0.037, *r* = −0.9), which suggests the decrease of type C tympanogram incidences with an increase in age. A particular correlation coefficient was obtained for the relationship between the amount of conductive problems cases and school grade of students at estimated value of *r* = −0.972, degree of freedom of *df* = 3, and confidence coefficient of *t* = 19.331, respectively. In other words, conductive problems are reduced with grade increase (Table 3).  Table 4 shows the results of conductive hearing loss prevalence in terms of gender. The difference was not statistically significant.  Table 5 shows the results of sensorineural hearing loss in terms of gender. The observed difference between the genders was not statistically significant. 

## 4. Discussion

The frequency of different hearing losses in this study was obtained as 8.8%. Therefore, compared with the study conducted in Bangladesh (11.9%) [9] and in Turkey (10.4% and 9.8%) [10, 11], a lower percentage of students in Zahedan had suffered hearing loss. However, the percentage of conductive hearing loss (7.9%) had a higher rate compared with that in Thailand (6.8%) [12] and India (4.79) [13] and also is very close to the study in Egypt (8.5%) [14]. Compared with a study conducted on primary school children in Australia, the rate of mild and minimal hearing loss was far more (8.2% versus 3%) [15]. The annual organized screening programs as well as greater public awareness and information can also be the reason of lower incidence of conductive hearing loss in a country like Australia [16]. 

The results of this study had some similarity with behavioral test results of Poland study [17]. In Iran, some researches were done, compared with a study conducted on primary schools of Tehran, in which the prevalence of different hearing losses was shown to be 14.3% [18]; in the present study, this rate was 8.8% for Zahedan. Similarly, a lower value was obtained for this rate compared with the value obtained for the cities of Birjand (10.4%) [19], Islamabad (9.7%) [20], and Mahabad (18%) [21]. In contrast, compared with studies conducted in the cities of Nishapur (5.5%) [22], Isfahan (4.2%) [23] and Shiraz (6.5%) [24], prevalence of hearing loss types was obtained higher in primary schools of the city of Zahedan. It is necessary to mention that the time gap between this study and other studies conducted in the country is at least ten years. Therefore, given the growing trend of preschool assessments in the whole country, it is expected that these figures have experienced decreasing trends in various cities. Overall, 20.2% of students in this study needed treatment measures for problems including excessive wax, type B and C tympanograms and external substance. Compared with studies conducted in other cities, the rate was only lower than the rate obtained in the study conducted in Tehran. Shortage of specialized personnel, notifications, and public awareness can be factors contributing to this problem; meanwhile, economic issues cannot be ignored with respect to timely action to overcome the hearing loss. The rate of students who were in need of rehabilitation measures was 0.9%, which shows less prevalence only compared with the rate obtained in the study conducted in Birjand. In this study, we were faced with limitations such as inadequate cooperation of parents for diagnostic evaluations—despite necessary follow-ups and free-of-charge examinations—in such a way that, from a total number of 354 students referred to in the first phase, 121 subjects left the study. Consequently, the statistic associated with the types of hearing losses is calculated based on the number of referrers. 

## 5. Conclusion

Hearing loss and its consequent difficulties on speech and language might be controlled and treated via appropriate hearing screening protocol and program in every educational setting; it is noteworthy that, according to the obtained results, the authors emphasize annual hearing screening programs for school-age children in order to promote health care and to prevent social and educational problems in this region, as these programs are being carried out in other areas. 

## Figures and Tables

**Table 1:** Results of direct observation and otoscopic examination based on gender.

Observation	Cerumen	External substance	Obstruction and narrowness of the canal	Eardrum rupture	Hearing aid
Male	65	4	1	2	4
Female	58	—	—	3	1

**Table 2:** Frequency distribution of types of tympanogram based on school grade.

Tympanogram	Grade											
	6		7		8		9		10		Total	
	Fr.	Percent	Fr.	Percent	Fr.	Percent	Fr.	Percent	Fr.	Percent	Fr.	Percent
A	243	81%	266	88.7%	267	89%	275	91.7%	275	91.7%	1326	88.4%
B	12	4%	10	3.3%	6	2%	6	2%	6	2.3%	41	2.7%
C	45	15%	24	8%	27	9%	19	6.3%	18	6%	133	8.9%

Total	300	100%	300	100%	300	100%	300	100%	300	100%	1500	100%

*Fr.: frequency.

**Table 3:** Frequency distribution of conductive hearing loss based on grade.

Conductive hearing loss	Grade											
	1st grade		2nd grade		3rd grade		4th grade		5th grade		Total	
	Fr.	Percent	Fr.	Percent	Fr.	Percent	Fr.	Percent	Fr.	Percent	Fr.	Percent
Yes	30	11%	25	9%	20	7.3%	20	7.3%	14	5%	109	88.4%
No	243	89%	252	91%	253	92.7%	254	92.7%	268	95%	1270	2.7%

Total	273	100%	277	100%	273	100%	274	100%	282	100%	1379	100%

**Table 4:** Frequency distribution of conductive hearing loss based on gender.

Conductive hearing loss	Gender					
	Male		Female		Total	
	Fr.	Percent	Fr.	Percent	Fr.	Percent
Yes	59	8.8%	50	7.1%	109	7.9%
No	611	91.2%	659	92.9%	1270	92.1%

Total	670	100%	709	100%	1379	100%

**Table 5:** Frequency distribution of sensorineural hearing loss based on gender.

Sensorineural hearing loss	Gender					
	Male		Female		Total	
	Fr.	Percent	Fr.	Percent	Fr.	Percent
Yes	7	1%	5	0.7%	12	0.9%
No	663	99%	704	99.3%	1367	99.1%

Total	670	100%	709	100%	1379	100%
